# Prophylactic anticoagulation in patients with glioblastoma or brain metastases and atrial fibrillation: an increased risk for intracranial hemorrhage?

**DOI:** 10.1007/s11060-021-03716-8

**Published:** 2021-03-05

**Authors:** Sina Burth, Mona Ohmann, Dorothea Kronsteiner, Meinhard Kieser, Sarah Löw, Lars Riedemann, Mona Laible, Anne Berberich, Katharina Drüschler, Timolaos Rizos, Antje Wick, Frank Winkler, Wolfgang Wick, Simon Nagel

**Affiliations:** 1 Department of Neurology, Heidelberg University Hospital, Im Neuenheimer Feld 400, 69120 Heidelberg, Germany; 2 Institute of Medical Biometry and Informatics, Heidelberg University Hospital, Im Neuenheimer Feld 130.3, 69120 Heidelberg, Germany; 3 Department of Neurology, Ulm University Hospital, Oberer Eselsberg 45, 89081 Ulm, Germany

**Keywords:** Intracranial hemorrhage, Glioblastoma, Metastasis, Anticoagulants, Atrial fibrillation

## Abstract

**Purpose:**

Patients with glioblastoma (GBM) or brain metastases (MET) and atrial fibrillation (AF) might be at an increased risk of intracranial hemorrhage (ICH) due to anticoagulation (AC). Our aim was to assess this risk.

**Methods:**

Our institution’s database (from 2005 to 2017) was screened for patients with GBM or MET and AF with an indication for AC according to their CHA_2_DS_2_VASc stroke risk score (≥ 2). Required follow-up was at least 3 months. AC was either performed with heparins, phenprocoumon or non-Vitamin K antagonist oral anticoagulants. Applying the propensity score approach, patient cohorts (matched according to primary tumor, age, sex) were generated (GBM [or MET] with AF ± AC, GBM [or MET] without AF/AC, no GBM [or MET] but AF on AC). ICH was defined as clinical deterioration caused by new blood on imaging. A log rank test was performed to compare the risk for ICH between the three groups.

**Results:**

In total, 104 patients were identified of which 49 with GBM (37% on AC) and 37 with MET (46% on AC) were successfully matched. Median follow up was 8.6 and 7.2 months, respectively. ICH occurred in 10.2% of GBM + AF and 12.2% GBM-AF, whereas 8% of patients with AF on AC suffered ICH (p = 0.076). 13.5% of patients with MET + AF had ICHs, in the controls it was 16% for MET-AF and 8% for AF on AC (p = 0.11).

**Conclusion:**

AC did not seem to influence the incidence of ICH in patients with glioblastoma or brain metastases within follow up of just under 9 months.

**Supplementary Information:**

The online version contains supplementary material available at 10.1007/s11060-021-03716-8.

## Introduction

Glioblastomas generally occur later in life with a median age at diagnosis of 65 years [1], whereas the incidence proportion for brain metastases varies depending on the primary tumor ranging between 20 and 39 years for breast cancer, 40–49 years for lung cancer and 50–59 years for melanoma, renal and colorectal cancer [2]. Elderly patients often have comorbidities that require management during cancer treatment. One is the need for anticoagulation (AC) to prevent systemic embolism due to atrial fibrillation (AF) which has been reported to be present in 1.4% of patients with glioblastoma [3]. With an increasing prevalence of AF with older age, 3.8% of people over 60 years suffer from AF and might be recommended to be anticoagulated due to a CHA_2_DS_2_VASc-Score, which identifies the risk of a person with AF to develop an ischemic stroke, of ≥ 2 [4].

However, anticoagulation in patients with brain tumors might put them at increased risk of intracranial hemorrhage (ICH) both in the perioperative period and in the subsequent phase of adjuvant treatment.

To date, most studies have examined the risk of ICH in the context of venous thromboembolism (VTE) which occurs frequently in patients with glioblastoma (15–30% of patients) [5–8] and brain metastases (four- to sevenfold higher risk [9]).

For patients with glioblastoma, anticoagulation is only administered in about 60% when indicated [6]. When anticoagulated for VTE, incidences of ICH vary between 2%, 7% and 4.7% in patients with glioblastoma in literature [3, 10, 11]. In one retrospective analysis patients (n = 133) with high-grade glioma on anticoagulation with enoxaparin were found to have a threefold increased risk of ICH (14.7% vs 2.5%) [12]. A recent meta-analysis confirms that especially patients with glioma have a threefold increased risk for ICH when on therapeutic anticoagulation for VTE [8]. Herein, the overall rates of ICH varied from 1.9 to 23% with a fatality rate of less than 1% [8].

In contrast, the risk for ICH is considered to vary for patients with brain metastases depending on the primary tumor. Patients with melanoma, choriocarcinoma, thyroid carcinoma, hepatocellular carcinoma and renal cell carcinoma were reported to be at particularly high risk [13, 14]. Recently, studies have found that anticoagulation did not influence the incidence of ICH for patients with metastatic melanoma, renal cell carcinoma or lung cancer [8, 15, 16], although the general risk was still fourfold increased for melanoma and renal cell carcinoma compared to lung cancer. According to another recent review, therapeutic anticoagulation in patients with treated brain metastases seems to have an acceptable risk of < 1% [17, 18].

In contrast to indications for therapeutic anticoagulation such as VTE, anticoagulation for AF is administered preventively to decrease the risk of stroke which is determined by the CHA_2_DS_2_VASc-Score. In this clinical scenario, stroke is only a potential, future risk but anticoagulation might increase the risk of bleeding in the present. Contrarily, a therapeutic anticoagulation for deep vein thrombosis or pulmonary embolism is inevitable as these conditions have to be treated despite the patients’ comorbidities. This study aims to assess the proportion of preventive full anticoagulation and the incidence of ICH by retrospectively analyzing two cohorts of patients with glioblastoma and brain metastases that also suffered from AF. Respective controls were cohorts of patients with glioblastoma or brain metastases without anticoagulation and of patients with AF on anticoagulation but without brain tumors.

## Methods

Inclusion of patients into this analysis is covered by a local Heidelberg ethics vote (No. S307/2019). To distinguish both a potential risk for ICH due to a brain tumor and the bleeding risk caused by anticoagulation we defined three groups. For the first group, our institution’s database (2005–2017) was screened for patients with a main diagnosis of a CNS tumor (ICD-codes C70.0 to C72.9) or CNS metastases (ICD-codes C79.3 to C79.4) that additionally suffered from AF (ICD-codes I48.0 to i48.9), were on continuous anticoagulation (ICD Z92.1) or had bleeding diatheses due to anticoagulation (ICD-codes D68.33 to D68.39). This group was named glioblastoma plus AF (GBM + AF) and metastases plus AF (MET + AF), respectively. The study was designed as an intention to treat study meaning that all of the patients in GBM + AF and MET + AF had an indication to be anticoagulated. The second group which was one of the control groups was acquired accordingly (2007–2017) using only the ICD-codes for glioblastoma (N = 628) or brain metastases (N = 434). This group ought to provide data on the baseline bleeding risk for patients with brain tumors that were not anticoagulated. The cohorts were called glioblastoma without AF (GBM-AF) and brain metastases without AF (MET-AF). Follow-up in all these groups was required until date of death or for at least 3 months. The third control group consisted of an already existing cohort of 90 patients from our institution with stroke or TIA with AF on anticoagulation [19] and a follow-up of 3 years. This group factors in the bleeding risk for patients that are anticoagulated but have no intracerebral neoplasm.

**Fig. 1:**
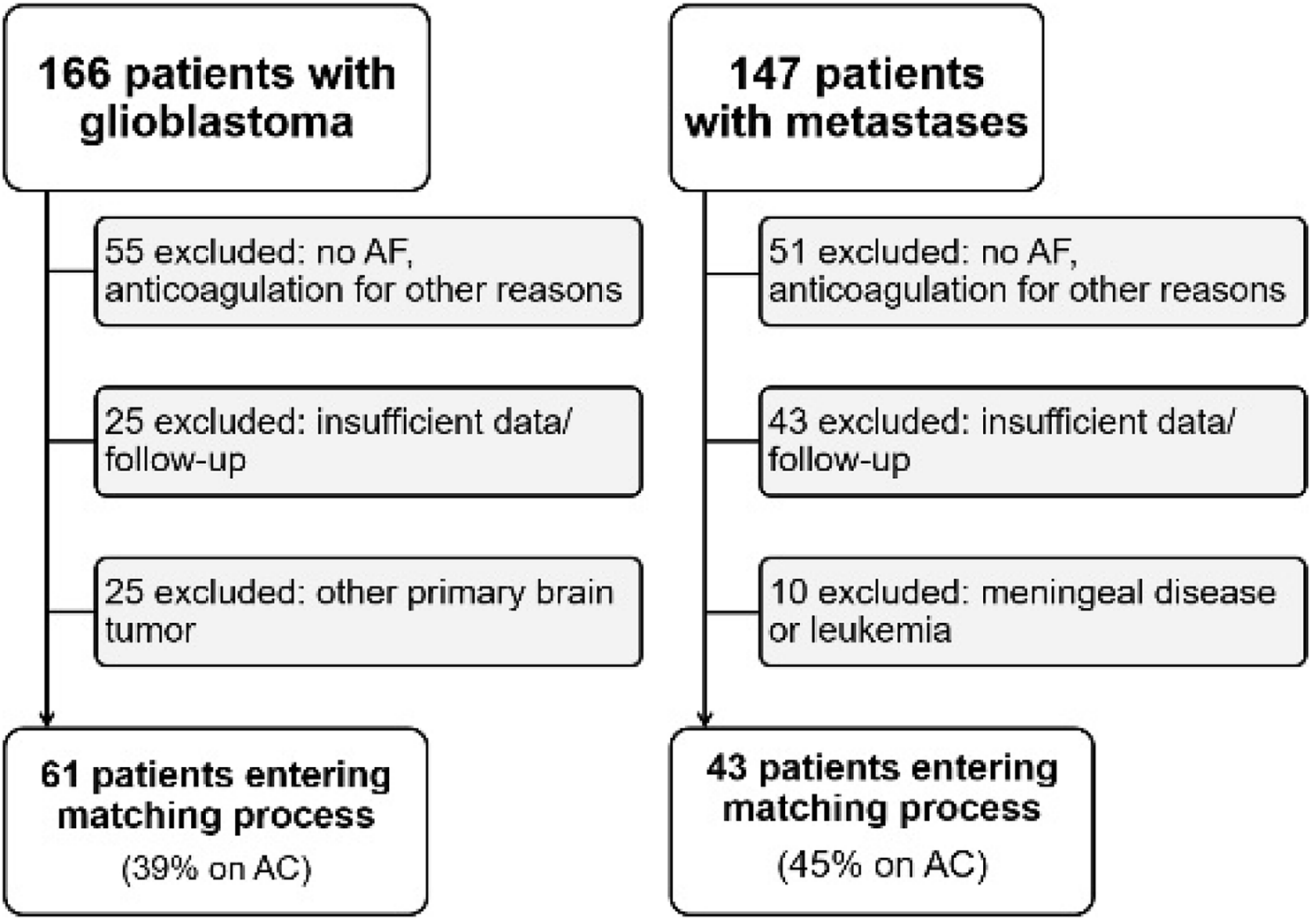
Selection process for patient cohorts. AC = full anticoagulation (receiving phenprocoumon, non-Vitamin K antagonist oral anticoagulants or at least enoxaparin 40 mg twice daily). *AF*  atrial fibrillation

From the initial screening, we identified 166 patients in the glioblastoma cohort (GBM + AF). Of these patients, 55 had to be excluded as they did not suffer from AF but were anticoagulated because of TVT, pulmonary embolism or other reasons. 25 patients had insufficient data or follow-up and 25 patients had a CNS tumor other than glioblastoma, leaving 61 patients that entered the matching process (Fig. 1).

Accordingly, 147 patients with brain metastases were identified (MET + AF). 51 patients were excluded as they were anticoagulated for reasons other than AF, 43 patients had insufficient data or follow-up and 10 patients had no solid metastases but suffered from meningeal disease or leukemia, leaving 43 patients that entered the matching process (Fig. 1).

**Fig. 2:**
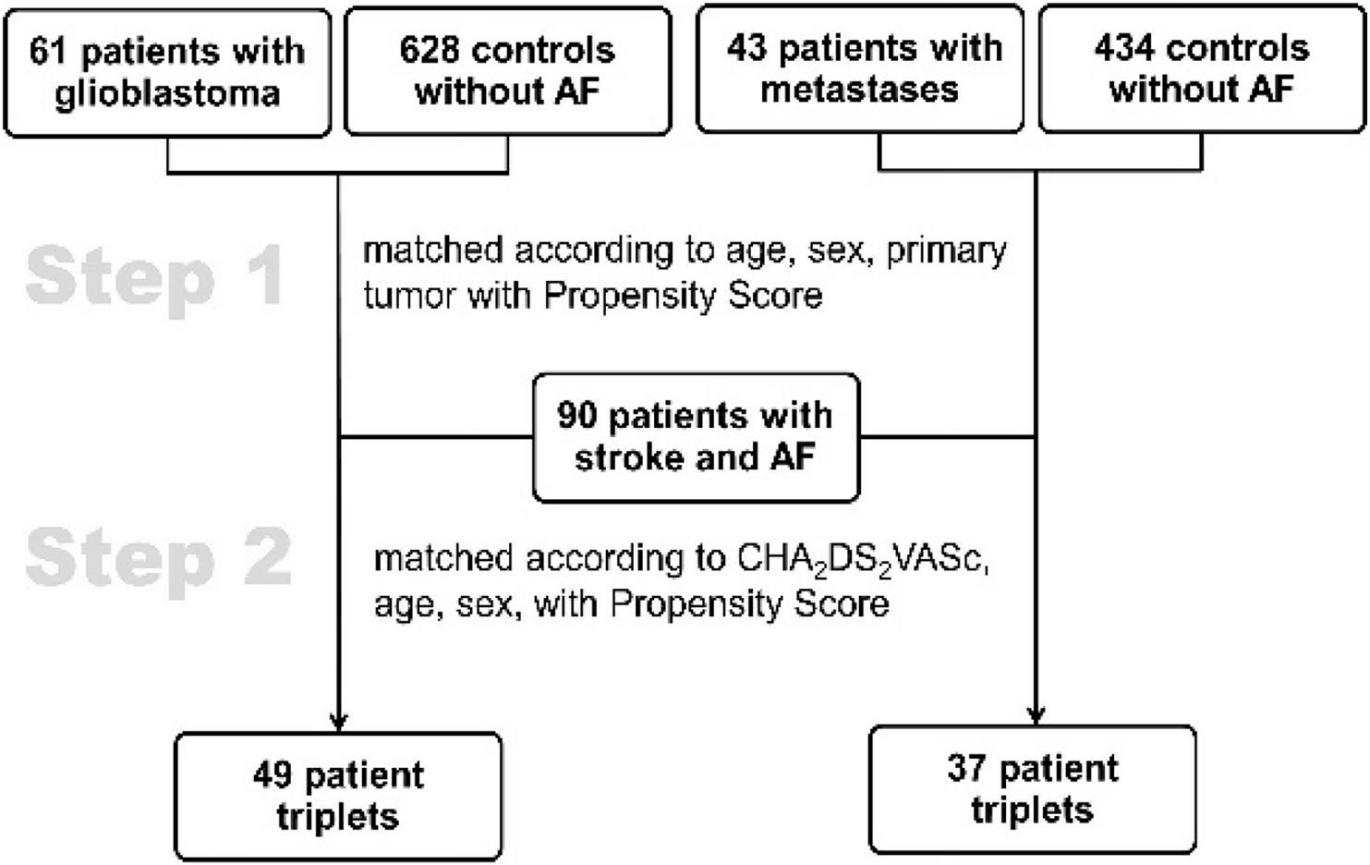
Matching process in 2 steps applying the propensity score. *AF* atrial fibrillation

A 2-step matching procedure was done using the propensity score approach [20]. The propensity score was estimated by logistic regression with group as dependent variable. The matching was done on the logit of the propensity score using a caliper of 0.2 of the standard deviation of the logit of the propensity score [20]. In a first matching step, 61 patients with glioblastoma (GBM + AF) and 43 patients with metastases (MET + AF) were matched to their control groups GBM-AF (N = 628) and MET-AF (N = 434) according to age, sex and primary tumor. In a second matching step, the vascular control group (stroke with AF on AC) was used yielding 49 triplets of patients for the glioblastoma group and 37 triplets of patients for the metastases group matched according to age, sex, primary tumor and CHA_2_DS_2_VASc-Score (Fig. 2).

**Fig. 3:**
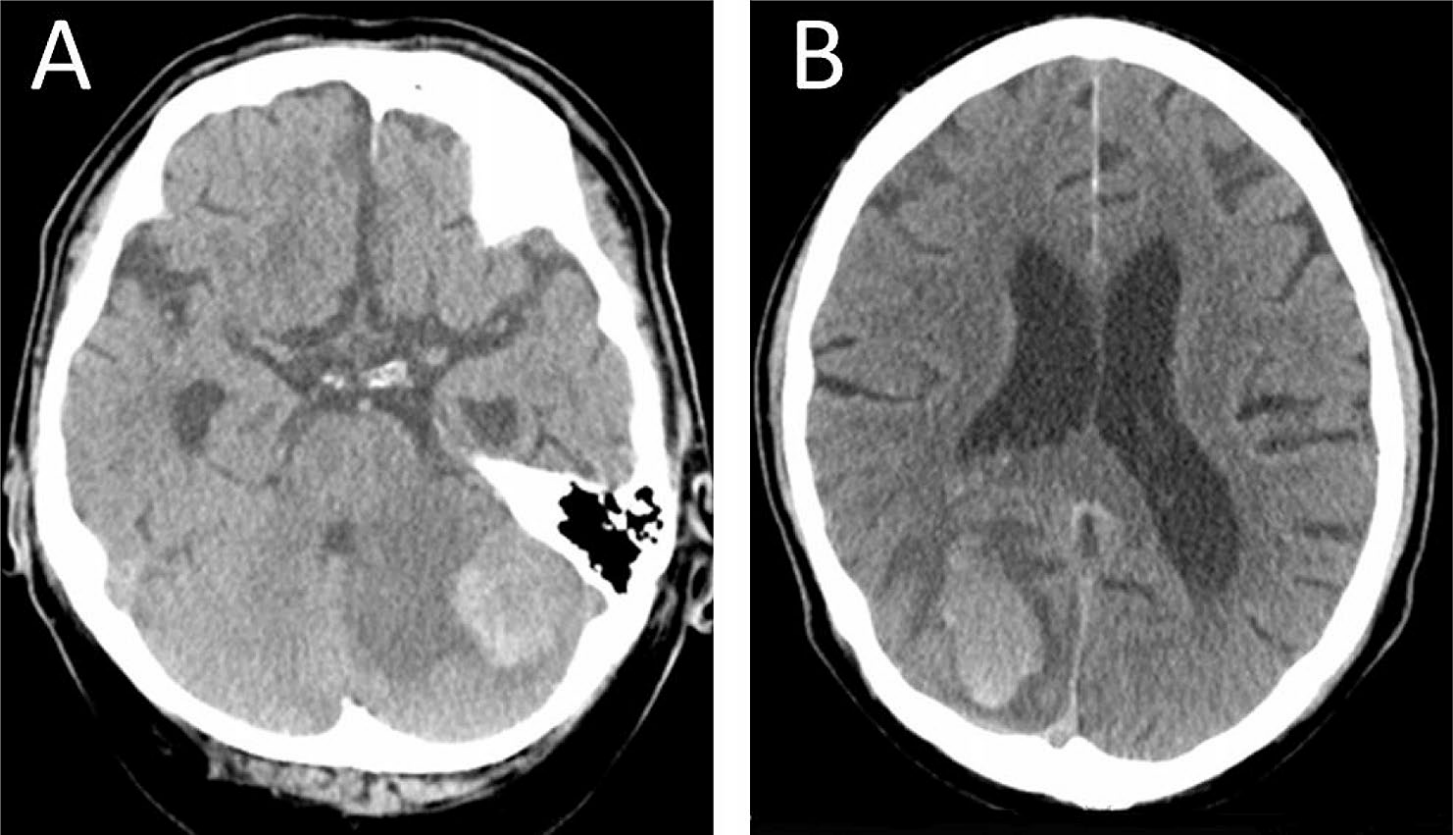
**a** Localized bleeding in a left cerebellar metastasis of a 63-year old male (lung cancer, on Rivaroxaban) presenting with a deterioration of gait. **b** Spontaneous right occipital ICH in a 74-year-old patient with melanoma. He presented with headache, nausea and vomiting and did not receive anticoagulation

For these patients, data was completed including age at diagnosis (defined as first detection of glioblastoma or brain metastases on imaging), sex, CHA_2_DS_2_VASc-Score, date of progression according to RANO criteria [21] and/or death (censored to the date of last follow up at an outpatient or inpatient presentation), methylation status of the MGMT-promoter and Karnofsky performance index where applicable, type of resection (biopsy, subtotal or total), adjuvant therapy regimen including chemotherapy, radiotherapy, both or palliative care and use of VEGF-receptor (vascular endothelial growth factor) inhibitors such as bevacizumab, which may increase the risk of bleeding [22], type and dose of anticoagulation and detailed information about bleeding and ischemic events. Full anticoagulation was defined as receiving phenprocoumon according to INR, receiving a non-Vitamin K antagonist oral anticoagulant or receiving heparin (dosage of at least 40 mg twice daily)*.* Prophylactic anticoagulation with a heparin (i.e. VTE prophylaxis) consisted of enoxaparin 40 mg once daily. Anticoagulation was documented if it was noted as a regular, long term medication in the patient’s admission/discharge medication plan. Bleeding events were primarily subclassified into microbleeds, locally contained or space occupying lesions (including midline shift or extension into the ventricles) as visible or described in the CT/MRI-reports (Fig. 3). Microbleeds were defined as punctate, homogeneous, rounded, hypointense lesions in the parenchyma that are smaller than 5–10 mm according to Kidwell, Wintermark et al. [23] and not counted as true bleeding events (slice thickness of our susceptibility weighted imaging was 2.5 mm). A separate category was included determining if the bleeding was clinically symptomatic with an altered level of consciousness, a new neurologic deficit, headache or nausea or warranted surgical treatment (“symptomatic”, sICH) or an incidental finding on routine imaging (“minor”), as the presence of contained, localized and small bleedings into a lesion would not be considered a harmful effect of the anticoagulation to the patient. Also, detailed follow-up imaging to detect such bleeds (in contrast to clinical information) was not available for all patients. Furthermore, the type of intracranial bleeding was noted (parenchymal, subdural, epidural, subarachnoid) and extracranial bleeding events were also documented (gastrointestinal etc.). Data was obtained as to whether the bleeding was present at the time of primary diagnosis or occurred perioperatively, spontaneous without anticoagulation or under anticoagulation. Ischemic events were subclassified into perioperative or spontaneous ischemic strokes, sinus thrombosis, pulmonary embolism, deep vein thrombosis or other extracranial ischemia such as bowel ischemia.

As the primary endpoint, the event of a clinically relevant symptomatic intracranial hemorrhage (sICH, yes/no) was chosen. Log-rank tests were performed to compare the groups concerning their bleeding events including the time until the event occurred to adjust for different follow-up times. For *post-hoc* (pairwise-) analysis of bleeding events over time Dunnett tests were used to adjust the p-values for multiple testing. Additionally, a mixed logistic regression was performed to examine the group (treatment vs. controls) as a possible explaining variable for the bleeding risk and using a random effect to take the matching procedure into account. Finally, McNemar tests were performed to compare the rate of strokes and bleeding events for patients with intracranial lesions and AF. Patient, disease, and therapy characteristics were analyzed descriptively using absolute and relative frequencies for categorical variables and median, first and third quartiles and range for continuous variables. Since this is an exploratory analysis p values are of descriptive nature. All statistical analyses were done using R version 6.3.3 [24].

## Results

The clinical characteristics of the cohorts are illustrated in Table 1. For the control group of patients with stroke and AF these were already published [19].

**Table 1:** Descriptive statistics of the main patient cohorts

Variable	GBM + AF (N = 49)	GBM-AF (N = 49)	Variable	MET + AF (N = 37)	MET-AF (N = 37)
Median age (range; q_1_/q_3_)	71 (37–84; 69/76)	70 (51–90; 67/74)	Median age (range; q_1_/q_3_)	71 (39–81; 63/74)	71 (33–83; 65/77)
Sex (male/female)	76%/24%	78%/22%	Sex (male/female)	67%/33%	67%/33%
Resection			Resection		
Biopsy	12 (24%)	4 (8%)	Yes	21 (57%)	20 (54%)
Subtotal	19 (39%)	12 (24%)	No	16 (43%)	17 (46%)
Total	18 (37%)	32 (65%)			
Adjuvant therapy			Adjuvant therapy		
Stupp	38 (78%)	33 (67%)	RT alone	22 (59%)	20 (54%)
Stupp + BEV	6 (12%)	15 (31%)	Chemo ± RT	10 (27%)	11 (30%)
Palliative	5 (10%)	1 (2%)	Palliative	5 (14%)	6 (16%)
Anticoagulation			Anticoagulation		
On full AC	18 (37%)	None		17 (46%)	None
NOAC	9 (18%)			6 (16%)	
Phenprocoumon	1 (2%)			2 (54%)	
Heparin	8 (16%)			9 (24%)	
Prophylactic AC/heparin	15 (31%)			7 (19%)	
None	16 (33%)			13 (35%)	
ICH			ICH		
Yes	8 (16%)	7 (14%)		5 (14%)	7 (19%)
No	41 (84%)	42 (86%)		32 (86%)	30 (81%)
Clinical presentation			Clinical presentation		
Silent (minor)	3 (6%)	1 (2%)		0	1 (3%)
Symptomatic (sICH) *(primary endpoint)*	5 (10.2%)	6 (12.2%)		5 (13.5%)	6 (16%)
Extend of bleed			Extend of bleed		
Local	2 (25%)	5 (71%)		3 (60%)	6 (86%)
Space-occupying	6 (75%)	2 (29%)		2 (40%)	1 (14%)
Localisation			Localisation		
Parenchymal	5 (20.0%)	6 (86%)		5 (100%)	6 (86%)
Subdural	2 (50.0%)	1 (14%)		0	0
Other intracranial	1 (30.0%)	0		0	1 (14%)
Extracranial	0	0		1	0
Surgical evacuation of ICH	4 (8%)	3 (6%)	Surgical evacuation of ICH	1 (3%)	1 (3%)
CHA_2_DS_2_VASc	3	/	CHA_2_DS_2_VASc	3	/
Median KPI	70	70	Median KPI	80	/
			Primary tumor		
			Lung	19 (51%)	20 (54%)
			Breast	5 (14%)	3 (8%)
			Melanoma	3 (8%)	6 (16%)
			Kidney	3 (8%)	1 (3%)
			Other	7 (19%)	7 (19%)

*AC* anticoagulation, *AF* atrial fibrillation, *BEV* Bevacizumab, *GBM* glioblastoma, *ICH* intracranial hemorrhage, *KPI* Karnofsky Performance Index, *MET* brain metastases, *N* number of patients per cohort, *NOAC* non-vitamin K antagonist oral anticoagulants, *q*_*1*_ first quartile, *q*_*3*_ third quartile, *RT* radiotherapy, *sICH* symptomatic ICH, *Stupp* therapy regimen according to Stupp et al. (Temozolomide plus radiation)

Five (10.2%) of patients with glioblastoma with AF suffered a sICH within a median follow-up time of 8.6 months (range 1–72 months). Two bleeds occurred under anticoagulation (40%), two perioperatively (40%) and one spontaneously without anticoagulation (20%). Looking at the different subgroups within the cohort of GBM + AF, N = 18 patients (37%) received full anticoagulation (i.e. Enoxaparin 40 mg 1-0-1, Phenprocoumon according to INR or a NOAC) and two sICHs occurred yielding a bleeding rate of 11%. In the subgroup that received anticoagulation for the prevention of VTE (i.e. Enoxaparin 40 mg 0-0-1, N = 15, 31%) or where anticoagulation was not administered or paused (N = 16, 32%) three sICHs occurred yielding a bleeding rate of 9.7%. 49% of patients died within the follow-up period. Median CHA_2_DS_2_VASc-Score was 3. Five patients suffered a stroke (10%), one patient a sinus thrombosis (2%).

In the control group without AF, six patients (12.2%, two spontaneously without anticoagulation and four perioperatively; 33% and 67%) had sICH within a median follow-up time of 14.9 months (range 7–97 months). The log rank test comparing all three groups revealed no difference of ICH between the three groups (p = 0.076), neither did the Dunnet test for the comparison of the groups glioblastoma with AF/without AF (p = 0.991) and glioblastoma with AF/vascular control group (p = 0.123) (Table 2, Kaplan–Meier curves are provided in Online Resource 1). In the logistic regression model the group did not seem to influence the event (p = 0.749 for GBM + AF vs. GBM-AF and p = 0.465 for GBM + AF vs. stroke with AF on AC). Hence, here anticoagulation had no effect on the incidence of ICHs in patients with glioblastoma. McNemar test showed no difference in the rate for bleeding events and strokes for patients with glioblastoma and AF (p = 1.0).

**Table 2:** Results of the Dunnet tests and the log rank test for patients with brain tumors and their control groups

Group comparison	Number of sICHs within the cohort	p value
GBM with AF (37% on AC) vs. GBM without AF and AC (control) *(Dunnet-test)*	5 (2 under anticoagulation) vs. 6	0.991
GBM with AF (37% on AC) vs. Stroke patients with AF on AC (control) *(Dunnet-test)*	5 (2 under anticoagulation) vs. 4	0.123
All three groups *(log-rank-test)*		0.076
MET with AF (46% on AC) vs. MET without AF and AC (control) *(Dunnet-test)*	5 (3 under anticoagulation) vs. 6	0.765
MET with AF (46% on AC) vs. Stroke patients with AF on AC (control) *(Dunnet-test)*	5 (3 under anticoagulation) vs. 3	0.253
All three groups *(log-rank-test)*		0.11

*AC* full anticoagulation, *AF* atrial fibrillation, *GBM* glioblastoma, *MET* brain metastases, *sICH* symptomatic intracranial hemorrhage

In the patient cohort with brain metastases and AF, a symptomatic ICH occurred in five patients (13.5%) within a median follow-up time of 7.2 months (range 0.5 to 143 months). Three bleeds (60%) occurred under anticoagulation, one with initial diagnosis (20%) and one spontaneously without anticoagulation (20%). Looking at the different subgroups within the cohort of MET + AF, N = 17 patients (46%) received full anticoagulation (i.e. Enoxaparin 40 mg 1-0-1, Phenprocoumon according to INR or a NOAC) and three sICHs occurred yielding a bleeding rate of 17.6%. In the subgroup that received anticoagulation for the prevention of VTE (i.e. Enoxaparin 40 mg 0-0-1, N = 7, 19%) or where anticoagulation was not administered or paused (N = 13, 35%) two sICHs occurred yielding a bleeding rate of 10%. 51% of patients died within the follow-up period. Median CHA_2_DS_2_VASc-Score was 3. Three patients suffered a stroke (8%), one patient additionally developed a pulmonary embolism (2.7%).

For the control group without AF incidence of sICH was 16.2% (total of 6 patients) within 8.4 months (range 0.25 to 62 months). Four bleeds occurred spontaneously (67%) and two with the initial diagnosis (33%). The log rank test comparing all three groups revealed no considerable difference of ICH between the three groups (p = 0.11), neither did the Dunnet test for the comparison of the groups brain metastases with AF/without AF (p = 0.765) and brain metastases with AF/vascular control group (p = 0.253) (Table 2, Kaplan–Meier curves are provided in Online Resource 2). In the logistic regression model the group did not seem to influence the event (p = 0.499 for MET + AF vs. MET-AF and p = 0.692 for MET + AF vs. stroke with AF on AC). McNemar test showed no difference in the rate for bleeding events and strokes for patients with brain metastases and AF (p = 1.0).

## Discussion

This study specifically addressed the risk/benefit assessment of anticoagulation for patients with brain tumors and AF. The existing literature primarily focuses on examining therapeutic anticoagulation for venous thromboembolism which has been shown to occur more often in patients with cancer [5–9]. While the latter is a therapeutic anticoagulation, anticoagulation for AF is prophylactic and one might hesitate more due to a perceived higher intracranial bleeding risk in GBM and MET patients.

Our data suggest that anticoagulation neither increases the risk for ICH in glioblastoma patients nor in patients with brain metastases within the given median follow up of 8.6 and 7.2 months. At least for the former group, a three-fold increased risk has been reported [8]. At 10.2% for the patients with glioblastoma and AF and 12.2% for the control group, the incidence of ICH lies within the reported range of 1.9–23% [8]. Our analysis was planned as intention to treat study, but since this was a retrospective analysis, it was not always clear why patients did not receive (oral) anticoagulation in the end. Still, the bleeding rates in the subgroups of patients on full anticoagulation were similar to these of the whole collective (11% vs. 10.2% for GBM + AF and 18% vs. 13.5% for MET + AF).

In the group GBM + AF, 12% of patients received bevacizumab, in the control group GBM-AF it was 33%. Due to the design of our study, we were not able to correct for a therapy with bevacizumab, which is known to increase the risk for bleeding complications [25]. For patients with brain metastases, our data is comparable to studies examining anticoagulation for venous thromboembolism. The proportion of ICH was slightly increased for patients with melanoma (accounting for 45% of all ICH) compared to their representation within the cohort (12% of patients had melanoma). The same effect was not observed for renal cell carcinoma. A possible confounding factor to the incidence of hemorrhage is the radiation protocol as hemorrhage is a known complication of stereotactic radiosurgery [26, 27]. In combination with immunotherapy or targeted therapy, radiosurgery is increasingly established as the standard of care and replacing whole brain radiotherapy for patients with brain metastases [28, 29]. However, as we could not perform a multivariate analysis, we cannot exclude confounding factors such as radiotherapy, the number and size of metastases, antiangiogenic therapy or immunotherapy on the incidence of ICH.

With a median CHA_2_DS_2_VASc-Score of 3, the risk of stroke is expected to be about 3% per year in our cohorts [30]. In fact, the incidence of stroke was as high as 10% in our study, probably because 54% (brain metastases) and 63% (glioblastoma) of patients were not sufficiently anticoagulated and because of the inherently higher risk of patients with cancer to develop arterial and venous thromboembolism [31].

Obvious limitations of our analysis are the retrospective nature which carries potential distortions. Firstly, a lot of the initially screened patients had to be excluded to fit the matching and follow-up criteria and due to missing data. Secondly, AC for AF was usually given per local standard if the individual presumed risk for bleeding was not increased. Thirdly, a matching process had to be implemented because the extraction of all available patient data would not have been feasible within a reasonable time frame and fourthly the median follow-up was just under 9 months. Still, the overall size of the cohort (N = 86) is acceptable as the largest reported cohorts to date included 133 and 107 patients [12, 16]. Furthermore, we managed to build well-matched cohorts using the Propensity Score and were able to also include a cohort of patients without brain tumors that were anticoagulated. Secondly, only a total of 37% of patients in the glioblastoma group and 46% of patients in the brain metastases group received full anticoagulation while 31% respectively 19% were on prophylactic anticoagulation. While this might weaken the effect of anticoagulation on the bleeding risk, it is also a valuable insight into the existing clinical practice with doctors hesitating to prescribe anticoagulation in patients with brain tumors although recent data suggests that this is safe at least for adequately treated brain metastases [17].

In conclusion, patients with both glioblastoma and metastatic brain tumors and AF did receive prophylactic anticoagulation in 37% and 46% in our large institutional database, most likely due to perceived increased risk of intracranial bleeding. In those patients that did receive prophylactic anticoagulation we could not demonstrate an increased incidence of ICH compared to patients with brain tumors and no anticoagulation or patients on anticoagulation that had no brain tumor. In GBM and MET patients with AF and high risk of embolic stroke a sufficient anticoagulation according to the CHA_2_DS_2_VASc-Score might still be warranted.

## Supplementary Information

Below is the link to the electronic supplementary material.

Electronic supplementary material 1 (DOCX 244 kb)

## Data Availability

The datasets generated during and analyzed during the current study are available from the corresponding author on reasonable request.
