# Supplementary material for: Prophylactic anticoagulation in patients with glioblastoma or brain metastases and atrial fibrillation: an increased risk for intracranial hemorrhage?
Source: J Neurooncol. 2021 Mar 5;152(3):483–90. doi: 10.1007/s11060-021-03716-8 (PMC8084835; doi:10.1007/s11060-021-03716-8)

**Online Resources**

**Online Resource 1** Corresponding Kaplan-Meier curve to the log-rank-test for the cohorts of patients with glioblastoma and their control groups comparing their event probabilities for intracranial hemorrhage. AC=anticoagulation, AF=atrial fibrillation, GBM=glioblastoma
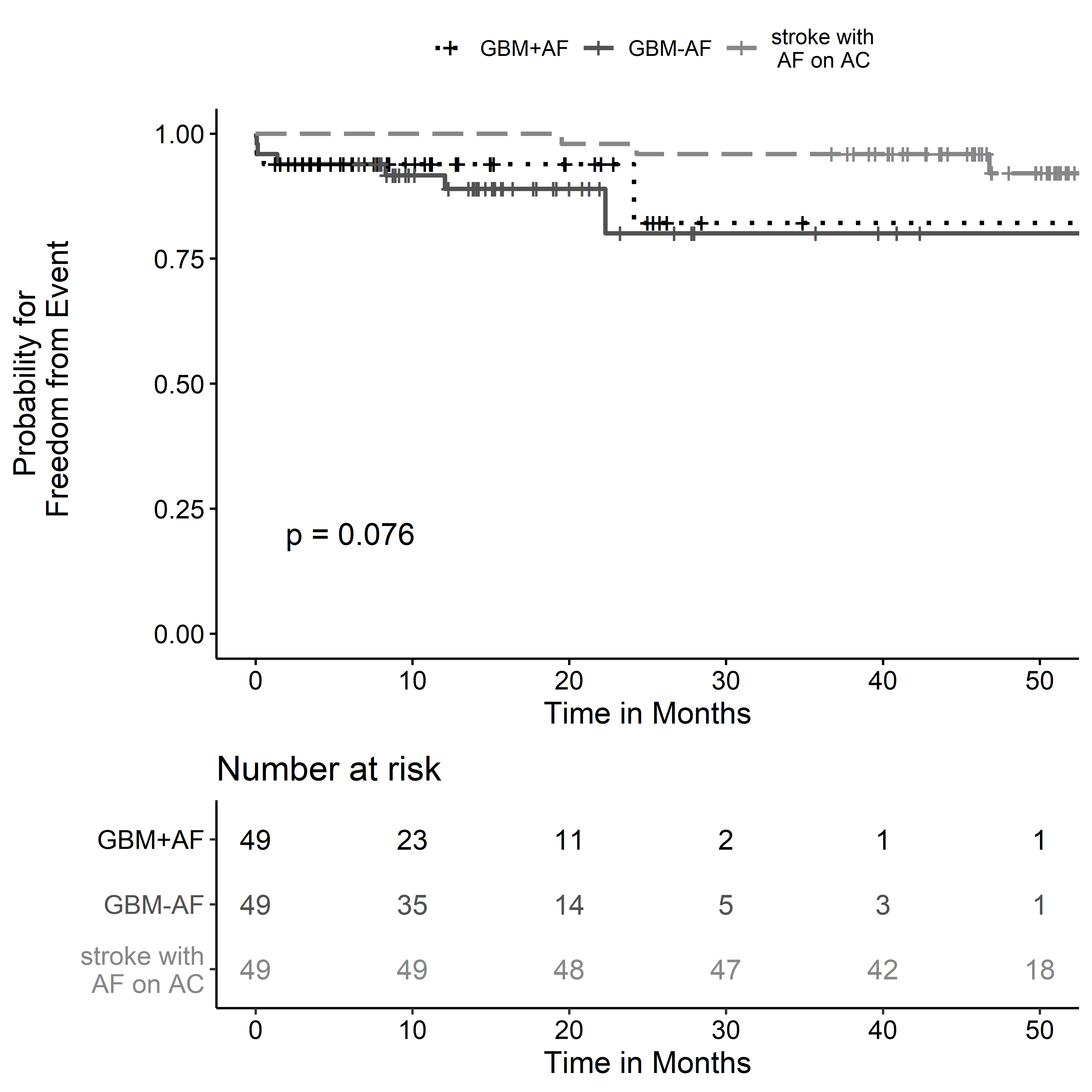


**Online Resource 2** Corresponding Kaplan-Meier curve to the log-rank-test for the cohorts of patients with brain metastases and their control groups comparing their event probabilities for intracranial hemorrhage. AC=anticoagulation, AF=atrial fibrillation, MET=brain metastases


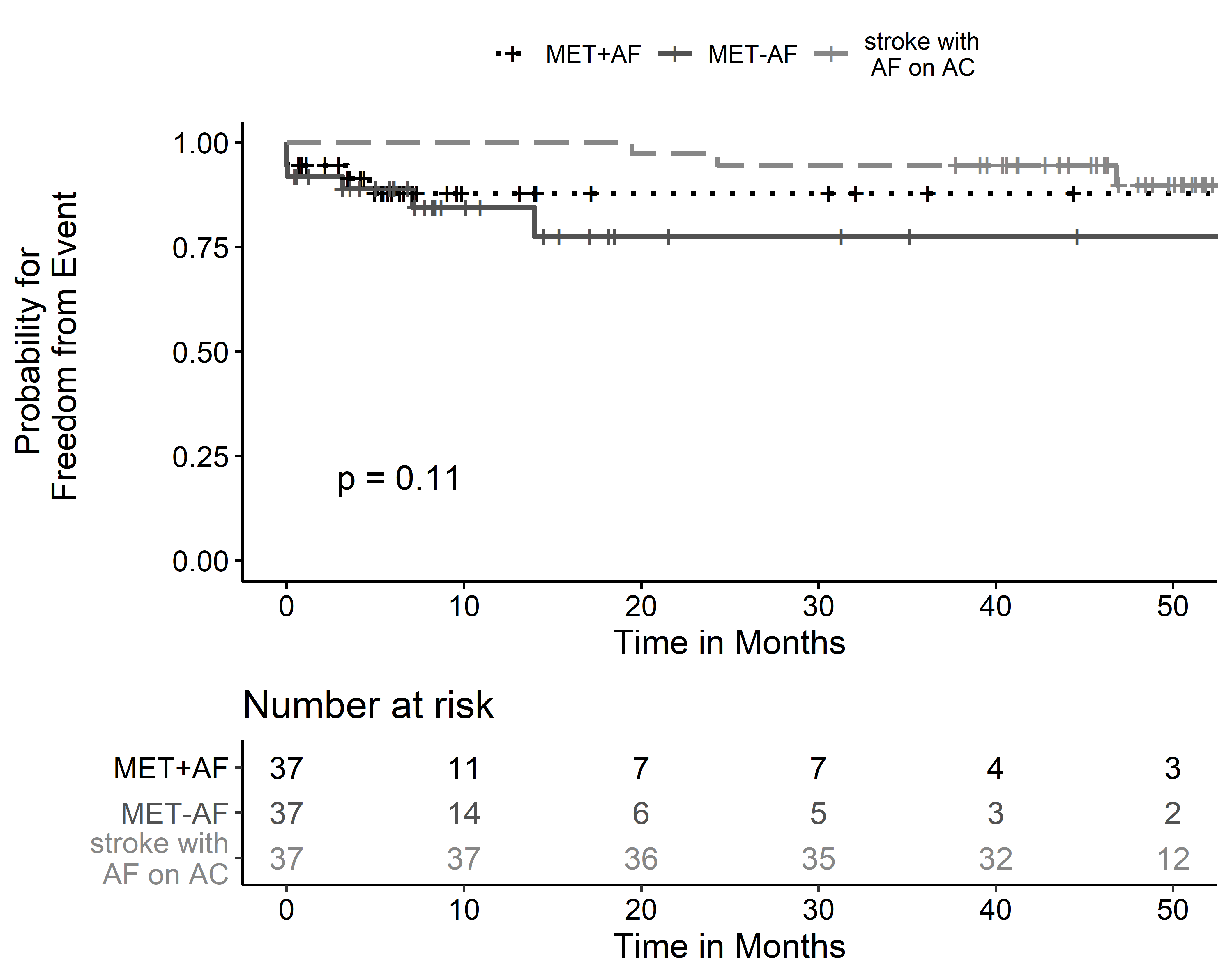

Supplement: Supplementary file 1 — Electronic supplementary material 1 (DOCX 244 kb) [file 11060_2021_3716_MOESM1_ESM.docx]
